# ICU-Associated Gram-Negative Bloodstream Infection: Risk Factors Affecting the Outcome Following the Emergence of Colistin-Resistant Isolates in a Regional Greek Hospital

**DOI:** 10.3390/antibiotics11030405

**Published:** 2022-03-17

**Authors:** Marios Karvouniaris, Garyphallia Poulakou, Konstantinos Tsiakos, Maria Chatzimichail, Panagiotis Papamichalis, Anna Katsiaflaka, Katerina Oikonomou, Antonios Katsioulis, Eleni Palli, Apostolos Komnos

**Affiliations:** 1 Intensive Care Unit, AHEPA University Hospital, 54636 Thessaloniki, Greece; 2 Third Department of Internal Medicine, School of Medicine, Sotiria General Hospital, National and Kapodistrian University, 11527 Athens, Greece; gpoulakou@gmail.com (G.P.); konstantinostsiakos@gmail.com (K.T.); 3 Intensive Care Unit, General Hospital of Larissa, 41221 Larissa, Greece; hatzimihail_m@yahoo.gr (M.C.); ppapanih@uth.gr (P.P.); kgoikonomou@hotmail.com (K.O.); komnosapo@gmail.com (A.K.); 4 Department of Microbiology, General Hospital of Larissa, 41221 Larissa, Greece; akatsaf@med.uth.gr; 5 School of Nursing, University of Thessaly, 41500 Larissa, Greece; akatsioul@med.uth.gr; 6 Intensive Care Unit, General University Hospital of Larissa, 41110 Larissa, Greece; kimnef@yahoo.gr

**Keywords:** APACHE II score, bacteremia, bloodstream infection, broth microdilution, colistin, colistin-resistant, Gram-negative, intensive care unit, mortality, SOFA score

## Abstract

Intensive care unit patients may present infections by difficult-to-treat-resistant Gram-negative microorganisms. Colistin resurfaced as a last resort antibiotic for the treatment of multi-drug-resistant Gram-negative bacteria. However, colistin might not improve survival, particularly after the emergence of colistin-resistant isolates. We aimed to (1) examine the first Gram-negative-associated-bloodstream infection (GN-BSI) effect on 28-day mortality and (2) distinguish mortality risk factors. From 1 January 2018 to 31 December 2019, we retrospectively studied all adult patients admitted for more than 48 h in the critical care department of a regional Greek hospital, with prevalent difficult-to-treat Gram-negative pathogens. We examined the patient records for the first GN-BSI. The local laboratory used broth microdilution to evaluate bacterial susceptibility to colistin. Seventy-eight patients fulfilled the entry criteria: adult and first GN-BSI. They developed GN-BSI on day 10 (6–18), while the overall mortality was 26.9%. Thirty-two and 46 individuals comprised the respective colistin-resistant and colistin-sensitive groups. The admission Acute Physiology Assessment and Chronic Health Evaluation II score was associated with acquiring colistin-resistant GN-BSI in the multivariable logistic regression analysis (οdds ratio (CI), 1.11 (1.03–1.21)). Regarding mortality, the index day sequential organ failure assessment score was solely associated with the outcome (hazard-ratio (CI), 1.23 (1.03–1.48), Cox proportional hazard analysis). GN-BSI was often caused by colistin-resistant bacteria. Concerning our data, sepsis severity was the independent predictor of mortality regardless of the colistin-resistance phenotype or empirical colistin treatment.

## 1. Introduction

Intensive care unit patients are predisposed to bacterial infection, as they are exposed to invasive devices and the critical illness might impair their immune response. A large worldwide point prevalence study of infections in the intensive care unit (ICU) found that 15.1% of the infected patients had bacteremia [1]. Another multicenter study highlighted that multi-drug-resistant Gram-negative (MDR-GN) bacteria are responsible for most bacteremic episodes and are associated with increased mortality [2]. Polymyxin E (colistin) is a drug of last resort to deal with these difficult-to-treat, often carbapenem-resistant, microorganisms [3]. Colistin is a polycationic peptide that disrupts the bacterial cell by binding to its anionic lipid A (endotoxin) part of the outer lipopolysaccharide membrane. The drug also possesses in vivo anti-endotoxin activity, and free radical generation through its passage via the outer bacterial membrane [4,5].

However, empirical colistin treatment may fail to demonstrate efficacy against carbapenem-resistant bacteria [6,7]. Meanwhile, acquired resistance to this drug has spread globally, following its increased use in agriculture and medicine. Bacteria present various genetic determinants of colistin resistance, either chromosomal or plasmid-related. Notably, the latter, transferrable plasmid-mediated resistance genes, can spread fast through the food chain. At the time being, their expanding list requires vigilant epidemiological surveillance [8]. Regarding resistance mechanisms, modification of the lipid A component of the outer bacterial membrane, via the chromosomal modulation of PmrAB and PhoPQ two-component systems, can lead to a decreased negative membrane charge, and, thus, to lower detergent action of the drug [4,5]. Moreover, bacteria may shed capsular polysaccharides that bind to colistin and decrease its availability to interact with the membrane molecules or may possess efflux pumps [4,5,9]. The increasing colistin-resistance prevalence can be particularly challenging in countries with an overall heavy MDR burden, infection control challenges, and submarginal antimicrobial stewardship [10]. Additionally, heteroresistance to colistin, i.e., a resistant subpopulation that co-exists as part of an otherwise sensitive population, may not allow the correct classification of the MDR-GN bacteria, regarding colistin susceptibility status [4]. Finally, the clinical interpretation of colistin resistance has been jeopardized by methodological issues on susceptibility testing. The European Committee On Antimicrobial Susceptibility Testing (EUCAST) recently issued guidelines (second version) for the detection of resistance mechanisms; the document advises laboratories to invariably use broth microdilution in the process of distinguishing colistin-resistant microorganisms to avoid major errors in the interpretation of susceptibility [11]. 

Although bloodstream infections (BSI) occur less often than lower respiratory tract infections [1], the isolation of a microorganism in a blood sample is solid evidence of infection compared to an isolate recovered from the tracheal secretions, which may represent colonization [12]. 

We aimed to explore the impact of a first episode of GN bacteremia on the primary outcome of 28-day all-cause mortality and other secondary endpoints. Additionally, we aimed to identify risk factors for (1) a colistin-resistant (CR) bacteremic episode and (2) 28-day mortality in an area of prevalent and endemic multi-drug resistance, after the adoption of EUCAST recommendations concerning colistin’s susceptibility testing. 

## 2. Materials and Methods

Our current study results were presented in part at the 40th International Symposium on Intensive Care and Emergency Medicine.

### 2.1. Study Design, Setting, and Selection Criteria

The study setting was a 16-bed mixed ICU in a regional hospital with 400 admissions per year. It is one of the largest ICUs in central Greece, an area populated by one million people. 

From 1 January 2018 to 31 December 2019, all adult patients with an ICU stay >48 h had their data retrospectively examined for the presence of a GN-BSI. 

The infection control policy comprises a hand hygiene protocol and widely recommended bundles concerning ventilator-associated pneumonia and catheter-related BSI prevention [13,14]. More specifically, to prevent CVC-related BSI, the bundle included the following measures: (1) meticulous hand hygiene, (2) insertion of CVC through echocardiographic guidance and with full-barrier precautions, (3) skin disinfection with chlorhexidine, (4) avoiding the femoral vein as a CVC placement site, and (5) disposal of nonessential CVCs. Whenever the CVC catheter had remained in place for more than 48 h and there was a suspicion of infection without an evident focus, it was removed. 

The protocol for culturing includes: (1) avoidance of routine culturing, (2) culturing whenever there is a suspicion of infection or sepsis, (3) at a minimum, we draw two sets of blood cultures, one from the central venous catheter if present and the other through venipuncture, (4) a single positive blood culture suffices for the diagnosis of GN-BSI, (5) the CVC tip is cultured after its withdrawal, and (6) routine lower respiratory tract culturing is performed through endotracheal aspirate sampling, to validate infection or once weekly for surveillance reasons. 

Regarding colistin administration, the individuals with normal renal function received 4.5 million units twice daily; otherwise, the dose was modified accordingly [15]. Patients in need of continuous renal replacement therapy were given a higher colistin dose of 6 million units bis in die [15].

This hospital’s microbiology department had adopted broth microdilution for colistin susceptibility assessment since October 2017 [16]. 

The patients enrolled in this study fulfilled the following criteria: adult, first bloodstream infection due to a GN pathogen. We excluded non-bacteremic patients, individuals with Gram-positive or fungal BSI, and those with an incomplete data file. 

The handling of individual patient data followed the Declaration of Helsinki and the current Health Insurance Portability and Accountability Act regulations [17]. No informed consent was required, as we used anonymized hospital data. We reported our results based on the Statement on Strengthening the Reporting of Observational Studies in Epidemiology [18]. The ethics committee of the hospital approved this study (Protocol 187/4-11-2019). 

### 2.2. Variables

Variables of interest on admission were: age, sex, Charlson Comorbidity Index [19], prior ICU stay during the previous 12 months, medical or surgical admission category, infectious disease status, presence o-immunosuppression, and receipt of antibiotic therapy in the last three months. Moreover, we evaluated the clinical severity on the day of admission with the Acute Physiology Chronic Health Evaluation II score (APACHE II) and the Sequential Organ Failure Assessment (SOFA) score [20,21]. Before the event, we reported the CVC status (CVC for at least 48 h) and antibiotic treatment with activity against Gram-negative bacteria. We documented the day of the BSI event, its timing (<48 h or ≥48 h from admission), the bacteremia source, and whether it was controlled within 24 h following the episode. Finally, on the index day, we recorded the Pitt bacteremia score and the fever or hypothermia status [22]. We assessed the severity of the index event with the SOFA score every 48 h from day 2 before the event until day 10 after the episode. We also recorded the maximum body temperature, white blood cell count, C-reactive protein, and procalcitonin at the aforementioned 48 h intervals.

### 2.3. Definitions

We defined GN-BSI whenever there was a positive blood culture for a GN microorganism, and the patient presented clinical and laboratory indices of infection. Index day was the day of collecting the first positive blood culture (index culture) that recovered a GN isolate. ICU-associated GN-BSI was further defined as the first bacteremic episode under two circumstances: (1) When the index culture was collected after two days in the ICU [23]; (2) We also included earlier onset events if the patients had been treated in an ICU during the previous year, as they likely continued to carry bacteria having similar resistance profiles [24]. In the case of a prior ICU stay, the patient should have been discharged from the ICU at least one month before the current readmission to be considered a first bacteremia event. Therefore, it was less likely to misclassify a bacteremia recurrence as a first event. We defined recurrent bacteremia, sepsis, and septic shock accordingly [25,26]. We examined only the first GN bloodstream infection.

The local laboratory categorized the bacteria isolated for susceptibility according to EUCAST criteria (version 7.0; 2017, EUCAST) [16]. Colistin-resistance was considered a minimal inhibitory concentration higher than 2 ng/mL, in line with EUCAST reports [16]. In the case of a polymicrobial BSI, we considered an event as colistin-resistant if at least one GN isolate was resistant to the drug. A minimal inhibitory concentration ≥8 ng/mL defined carbapenem (meropenem) resistance according to the EUCAST clinical breakpoints [16].

Primary BSI, source control, and CVC-related BSI have been defined accordingly [27,28]. The empirical treatment delivered to an infected patient was considered appropriate if the drug(s) was (were) active in vitro to the isolate (or both isolates, if present) [29]. We defined immunosuppression in keeping with predefined criteria [21]. Renal failure was characterized as risk, injury, failure, loss, and end-stage kidney disease (RIFLE) stage ≥3 (with a 3-fold rise in the serum creatinine, urine volume less than 0.3 mL/kg/h for 24 h, or no urine output for 12 h, or the use of renal replacement treatment) [30]. 

### 2.4. Outcome

The primary study outcome was 28-day mortality after the event. Secondary outcomes were 14-day mortality post-event, overall ICU mortality, hospital mortality, ICU stay post-event, overall ICU stay. More secondary outcomes included recurrent bacteremia, secondary bacteremia, mechanical ventilation days post-event, overall mechanical ventilation days, renal failure-free days, renal SOFA at 7 and 14 days, and continuous renal replacement therapy at 7 and 14 days following the index day.

### 2.5. Statistical Analysis

We used the median with interquartile range (IQR) and number with percentage (%) to describe quantitative data and qualitative data, where appropriate. Fisher’s exact (or Chi-squared test) and Mann–Whitney test (or *t*-test) were used to compare qualitative and quantitative variables. Statistical significance was set at a *p*-value of <0.05. 

Missing data, concerning only laboratory values, were handled in keeping with a reported algorithm [31]. Specifically, we imputed missing numerical values with the respective median if the percentage of not-available numbers was <10%. However, we added the values derived from multivariate imputation, with the predictive mean matching method, if the total non-available variable values were less than 50%. Otherwise, we excluded the variables implicated.

The longitudinal variables were analyzed by comparing their means with the Tukey test and the alternative method “less”.

Regarding multivariable regression analyses [32], at first, we considered the clinical value of a variable before its inclusion into the model, regardless of the univariate comparison. Despite any significant difference, we have not included variables that did not convey unique information in the models assessed (i.e., immunosuppression status is included in the APACHE II score, the index day temperature is part of the Pitt bacteremia score, and event day septic shock status adds 4 points to the index day SOFA score). Secondly, we included any variable presenting a *p*-value of ≤0.10. Finally, we tested multicollinearity using a variance inflation factor score before inserting any variables in the model.

Overall, the optimal cutoffs of quantitative explanatory variables were assessed with the Youden criterion. 

We performed multivariable logistic regression analysis to evaluate risk factors for developing colistin-resistant BSI. The initial full model for CR phenotype was comprised of age, admission due to infection, Charlson Comorbidity Index, and the APACHE II and SOFA scores on admission. The final model was selected in a backward, stepwise method following a bootstrap resampling of the original data. The derived variables then entered the final model.

Regarding 28-day mortality, we used Kaplan–Meier survival analysis and log-rank test to assess the association between colistin susceptibility and mortality [33]. We also performed Cox proportional hazard analysis to evaluate time to 28-day mortality [33]. The initial, full model included age, Charlson Comorbidity Index, APACHE II, Pitt Bacteremia score, index day SOFA score, CR status, and empirical receipt of colistin for 5 days. The full model variables were examined for violation of proportionality assumption via the global Schoenfeld test and the visual inspection of the covariate Cox model plots. Any violating variable was used as a stratification variable. Afterward, the qualifying variables were regularized by the Least Absolute Shrinkage and Selection Operator to select the explanatory variables of the final model. Finally, the discriminative power of the final Cox proportional hazard model was evaluated with the concordance index. 

We also conducted three more statistical sensitivity studies: (1) regarding a threshold of colistin sensitivity at a minimal inhibitory concentration of 0.5 ng/mL instead of the recommended 2 ng/mL, (2) data were reanalyzed after exclusion of eleven patients who presented early BSI, before a 48 h stay in the ICU), and (3) finally, dividing the patients into two groups by the median SOFA score at the index day (the sicker group had a score ≥ 7).

We analyzed data with R version 4.0.3 (R Core Team, Vienna, Austria) [34].

## 3. Results

### 3.1. Population

**Figure 1:**
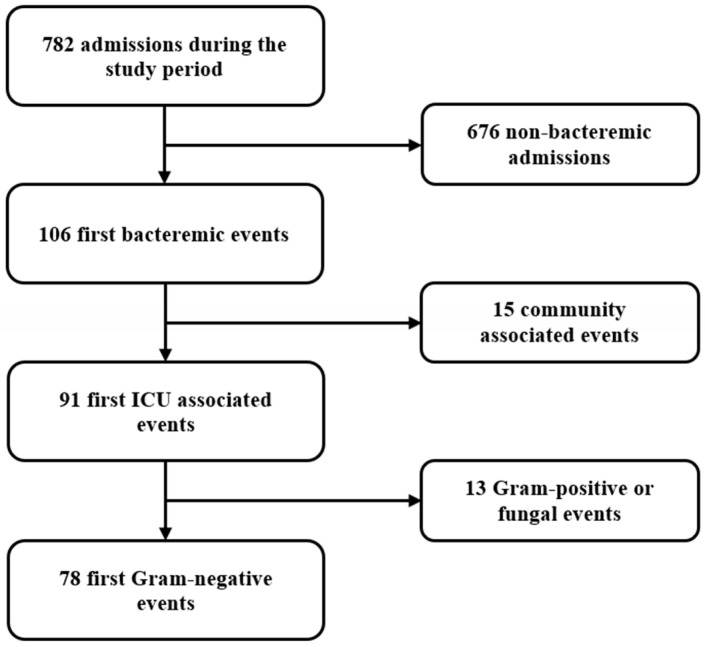
Study flowchart.

During the study period (Figure 1, flowchart), seventy-eight patients fulfilled the entry criteria: eleven (14.1%) received BSI diagnosis during the first two days of admission and the rest afterward. The colistin-resistant (CRG) and the colistin-sensitive groups (CSG) comprised thirty-two and forty-six patients, respectively. The baseline patient characteristics are shown in Table 1. Notably, most patients had received antibiotics in the three months before the admission, and a third had been treated in an ICU during the previous year. Before the event, 80% of the study participants had had a CVC in place and had received antibiotics. 

### 3.2. Infection

Bloodstream infection occurred on day 10 (IQR 6–18), and it was most often primary or related to intravascular catheter use (Table 1). Most isolates (73.1%) were carbapenem-resistant. Eight episodes were polymicrobial (including two GN isolates). The culprit isolates differed between groups; of note, the CRG included pathogens endogenously resistant to colistin (*Serratia* and *Providencia* spp.), and no *Pseudomonas* isolates, in contrast to the CSG (Table 2).

Concerning the colistin susceptibility phenotype, patients of the CRG were older and presented increased APACHE II score on the day of admission compared to the CSG; however, in the final multivariable logistic regression model, only the APACHE II score remained independently associated with the development of CR-GN-BSI (Table 1, Supplementary Tables S1 and S2).

The estimated optimal cutoff value of the APACHE II score for discriminating CR from CS events was 20, with an AUC (95% CI) of 0.67 (0.55–0.79), a sensitivity of 59.4%, and a specificity of 69.6%.

The clinical and laboratory parameters on the index day are displayed in Table 1; there were no significant differences between the two groups. Overall, septic shock was evident in 55.1% of cases. The presence of septic shock and the sepsis rate of the CRG, indicated by the SOFA score, were similar to the CSG (Supplementary Materials Figure S1).

The temporal evolution of the SOFA score, the maximum daily temperature, white blood cell count, C-reactive protein, and procalcitonin unveiled a limited, though steady decline (Supplementary Materials Table S1, *p* = 0.01). In the vast majority of measured values, there was no difference as to colistin susceptibility status (Supplementary Materials Table S3).

### 3.3. Sensitivity Analyses for the Occurrence of the Colistin-Resistant Phenotype

We re-explored the data by lowering the susceptibility threshold to a minimal inhibitory concentration to colistin of 0.5 ng/mL and confirmed the significance of the admission APACHE II score. Moreover, we found that ICU admission due to infection was found more often in the optimal colistin-sensitive group (Supplementary Materials Table S1).

We also repeated the analysis after the exclusion of eleven patients who presented early BSI, before a 48 h stay in the ICU. The reanalysis showed that the APACHE score was the sole independent variable associated with the presence of colistin BSI phenotype (Supplementary Materials Table S2).

### 3.4. Treatment

The infection was empirically, appropriately treated in less than half the cases (Supplementary Materials Table S4). The most commonly administered antibiotics, possessing anti-Gram-negative activity and used for the treatment of various infections before the BSI diagnosis, were: carbapenems in 33 (42.3%), colistin in 28 (35.9%), cephalosporins in 26 (33.3%), tigecycline in 24 (30.8%), and ampicillin-sulbactam in 15 (19.2%) patients. Colistin has already been given for a median of 12.5 days before the event (IQR 5–15.5 days). 

Thirty patients (45.5%) received combined targeted treatment for the GN-BSI event. The most frequently prescribed antimicrobial was colistin (30/67), followed by tigecycline (22/67), carbapenems (18/67), and aminoglycosides (16/67). 

Overall, colistin was extensively used, usually in combination with other drugs (Supplementary Materials Table S4). There was no difference between the two groups regarding treatment aspects; of note, at least five-day colistin administration in the CRG started as an empirical antimicrobial regimen was similar to the CSG. However, the CR individuals tended to receive delayed targeted therapy than the CSG (on day three vs. day 0).

### 3.5. Outcomes

The study outcomes are displayed in Supplementary Materials Table S5. 

**Figure 2:**
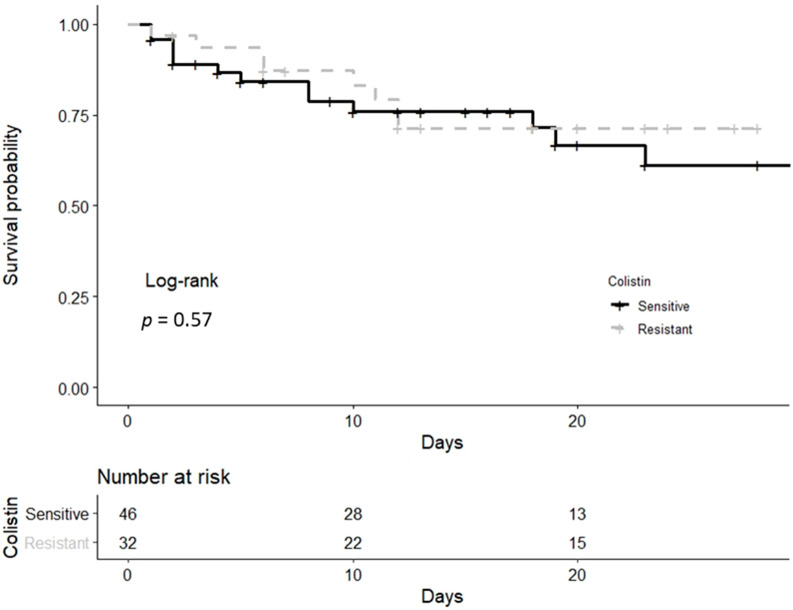
Kaplan–Meier survival curve of both colistin-resistant and colistin-sensitive groups until day 28 post-event.

Twenty-eight-day mortality post-event was overall 26.9%, and the Kaplan–Meier curves did not reveal any difference between the colistin-sensitive and the colistin-resistant groups (log-rank test, *p* = 0.57) (Figure 2). The corresponding 28-day mortality of the carbapenem-resistant *Acinetobacter baumannii* and *Klebsiella pneumoniae* infected patients was 34.5% and 28.6%.

Regarding 28-day mortality, univariate analysis and the hazard ratios of the multivariable Cox proportional hazard analysis are presented in Table 3. The SOFA score on the index day was independently associated with higher mortality (Table 3 and Supplementary Materials Table S5). The optimal discriminative cutoff value for the index day SOFA score was 11 (AUC (95% CI) 0.871 (0.77–0.97)), while the respective sensitivity and specificity were 76% and 88%.

Concerning sepsis evaluation, analysis of the data by using the median SOFA score threshold, which is valued at 7 in this dataset, the high SOFA score group had similar CR-BSI incidence compared to the lower SOFA score group. Of interest, the individuals with the higher score had independently had a prior ICU admission, increased Charlson Comorbidity Index, and Pitt bacteremia score on the event day (Supplementary Materials Table S7).

Recurrent bacteremia occurred on 8 (6–12) and secondary BSI on 12 (7–18) days following the index culture (Supplementary Materials Table S6). Secondary isolates were mostly Gram-negative (89%); the latter were often colistin-resistant (41.2%). Neither the primary analysis nor the alternative, using a strict, 0.5 ng/mL, the threshold for susceptibility to the drug, have revealed significant differences in the secondary study outcomes (Supplementary Materials Tables S5 and S8).

## 4. Discussion

The present study reports on a population of critically ill patients with GN-BSI presenting overall 28-day mortality of 26.9%. The occurrence of the colistin-resistant phenotype was independently associated with the patients’ clinical severity status on ICU admission, evaluated by an increased APACHE II score, and not with the antimicrobials administered. Similarly, the sepsis severity status of the patient on the index day, as assessed by the SOFA score, was associated with worse 28-day mortality; however, we could not link the colistin susceptibility status or administration of colistin to the outcome. 

In other studies, regarding CR, *K. pneumoniae*, and *A. baumannii*, infections had not presented increased admission severity in the non-susceptible group. However, their participants were often not critically ill and not exclusively bloodstream-infected [7,35]. Apart from the worse admission status, the CRG’s event SOFA score was higher, though not significantly, than the respective CSG’s value (*p* = 0.07). Re-analyzing the data by the index day SOFA value, the sicker patients (score ≥ 7) had independently had a prior ICU admission (Supplementary Materials Table S7), which is in line with a CDC-affiliated study showing that prior hospitalization with broad-spectrum antimicrobials’ exposure increases sepsis risk [36].

A recent ICU study showed that combined *A. baumannii* CR-BSI and septic shock were always fatal [37]. Only half of our population presented septic shock, and the corresponding mortality was in comparison lower, at 34.5%. However, according to the above, a higher event SOFA score was independently associated with 28-day mortality regardless of the CR phenotype (Table 3). Notably, a re-analysis of *A. baumannii*-infected patients unexpectedly found that the CR individuals presented less mortality than their CS counterparts [7]. Regarding combined carbapenem-resistant and CR *K. pneumoniae* infections, we observed a fatality rate of 28.6%, contrary to recent literature, which had exhibited increased mortality, over 50%, before ceftazidime-avibactam’s inception [35,38]. In this study, carbapenem-resistant *K. pneumoniae*-associated BSI patients often received ceftazidime-avibactam, as part of empirical or targeted treatment (data not shown), a drug with superior efficacy compared to colistin [39]. 

Colistin has recently re-emerged as therapy for the difficult-to-treat GN pathogens [3,40]. Regardless of the susceptibility status, colistin’s BSI treatment failed to add any survival benefit despite its extensive empirical use and its recommended dosing [14]. However, antimicrobial coverage’s appropriateness throughout the study groups was less than 50% in the first 48 h post-event (Supplementary Materials Table S4). Many CRG patients regularly received colistin as an empirical regimen, and they would likely have survived regardless of an ineffective antibiotic scheme. Unfortunately, similar to other investigators [6], we could not demonstrate any benefit from the empirical regimen. The reasons for the lack of colistin’s therapeutic efficacy could be the gloomy evolution of high-level resistance, leaving little room for efficacious antibiotic therapy, or the insufficient activity of the drugs delivered, notably colistin, or even the decreased fitness—virulence of the CR bacteria [41,42]. A final issue could be the possible antagonistic rather than synergistic effects of colistin with other antimicrobials, which may have influenced the outcome [40].

Nonetheless, there are in vitro data that seem promising for the development of future therapeutic strategies. At first, *Enterobacterales* bacterial strains that expressed the mobilized colistin-resistant gene-1 were tested for resistance to several antibiotics; these strains remained susceptible to eravacycline, which can be studied in vivo for the treatment of CR bacteria [43]. Analysis of the secondary resistome, i.e., genes that are not known resistance determinants, of *K. pneumoniae* has found a conditionally essential gene for the CR phenotype (only in the presence of colistin) [44]. That chromosomal gene encodes a DedA family membrane transporter protein, which can restore sensitivity to the drug if depolarized [45]. 

The study’s strength lies in the adoption of broth microdilution, a robust methodology concerning colistin susceptibility. The routine use of the recommended laboratory method minimizes bacterial misclassification and enables between-study comparison. Moreover, we dosed colistin according to the latest pharmacokinetic data [15]. Notably, we also included very early infections in those patients who had previously been cared for in an ICU. These patients probably remain critically ill, viewed from a microbiological viewpoint, as they carry resistant microbiota, which may evolve to even more resistant phenotypes through rehospitalizations [24,46].

The single-center study design limits its generalizability. Moreover, the investigation setting presents extreme multi-drug-resistant GN flora that renders the results plausible only to critical care departments with isolates of similar susceptibility patterns. In addition, we do not have local data regarding the molecular determinants of colistin resistance; however, it is likely to represent similar mutations as those reported from other Greek hospitals [47,48]. Another drawback is that the hospital laboratory had not performed assays to evaluate colistin’s synergy with other antibiotics; however, such assays are complex and of questionable predictive value for therapeutic efficacy [49].

## 5. Conclusions

ICU-associated Gram-negative bloodstream infection in a setting of limited treatment options can adversely impact outcomes. The colistin-resistant phenotype was more common in association with a high APACHE II score on admission. The higher SOFA score on the BSI index day was associated with increased 28-day mortality, contrary to the isolate’s susceptibility status to colistin or treatment of the episode with colistin, which were unassociated with this outcome. However, due to the study’s retrospective design, these observations should be re-evaluated in a future prospective study.

## Figures and Tables

**Table 1:** Patient characteristics on admission, before the bloodstream infection, and on the day of the event.

	Overall (*n* = 78)	Colistin-Resistant (*n* = 32)	Colistin-Sensitive (*n* = 46)	*p*-Value **	Odds Ratio (95% CI) ##
On admission					
Age, years	66 (50.2–76)	72 (59–78)	62.5 (47.7–74.5)	**0.07**	
Male	51 (65.4)	22 (68.7)	29 (63)	0.64	
Charlson Comorbidity Index	3 (1–5)	4 (2–5)	2.5 (1–5)	**0.25**	
APACHE II	19 (13–24)	21.5 (15.2–25)	17.5 (10.7–21)	**0.01**	1.11 (1.03–1.21)
SOFA score	8 (5–10)	9 (6.2–10)	7 (3.5–10.5)	**0.15**	
Prior ICU stay, previous year	27 (34.6)	12 (37.5)	15 (32.6)	0.80	
Medical patients	48 (61.5)	23 (71.9)	25 (54.3)	0.16	
Immunosuppression	9 (11.5)	6 (18.7)	3 (6.5)	0.14	
Admission due to infection	21 (26.9)	6 (18.7)	15 (32.6)	**0.20**	0.35 (0.1–1.07)
Antibiotics in the previous 3 months	48 (61.5)	19 (59.4)	29 (63)	0.64	
**Before the event**					
CVC for at least 48 h	61 (78.2)	25 (78.1)	36 (78.3)	>0.99	
Antibiotics in the ICU	61 (78.2)	24 (75)	37 (80.4)	0.59	
Maximum number of drugs with AGNA at any time				0.29	
None given	17 (21.8)	8 (25)	9 (19.6)		
Single	31 (39.7)	9 (28.1)	22 (47.8)		
Two	9 (11.6)	3 (9.4)	6 (13)		
Three	10 (12.8)	5 (15.6)	5 (10.9)		
Four	11 (14.1)	7 (21.9)	4 (8.7)		
Antibiotic classes/class members *					
Third & fourth generation cephalosporins	26 (33.3)	11 (34.4)	15 (32.6)		
Colistin	28 (35.9)	14 (43.8)	14 (30.4)		
Tigecycline	24 (30.8)	13 (40.7)	11 (23.9)		
Carbapenems	33 (42.3)	14 (43.8)	19 (41.3)		
Aminoglycosides	10 (12.8)	5 (15.6)	5 (10.9)		
Quinolones	13 (16.7)	10 (31.2)	3 (6.5)		
Ampicillin/sulbactam	15 (19.2)	8 (25)	7 (15.2)		
Piperacillin/tazobactam	9 (11.5)	4 (12.5)	5 (10.9)		
Ceftazidime/avibactam	7 (9)	4 (12.5)	3 (6.5)		
**Index day**					**NA**
Event, days	10 (6–18)	12 (5.2–21.5)	9.5 (6–17.2)	0.66	
Timing of the event				0.34	
>48 h stay	67 (85.9)	26 (81.3)	41 (89.1)		
<48 h stay	11 (14.1)	6 (18.7)	5 (10.9)		
Source				0.73	
Primary	32 (41)	13 (40.6)	19 (41.3)		
Catheter-related #	25 (32.1)	12 (37.5)	13 (28.3)		
Urinary	5 (6.4)	3 (9.4)	2 (4.3)		
Intraabdominal	5 (6.4)	1 (3.1)	4 (8.7)		
Surgical site infection	5 (6.4)	1 (3.1)	4 (8.7)		
Lung/pleural empyema	4 (5.1)	1 (3.1)	3 (6.5)		
Bone/joint	2 (2.6)	1 (3.1)	1 (2.2)		
Source control performed	30 (38.5)	15 (46.9)	15 (32.6)	0.24	
Pitt bacteremia score	3 (1–4)	4 (2–4.7)	3 (1–4.2)	0.25	
Septic shock	43 (55.1)	18 (56.2)	25 (54.3)	>0.99	
Temperature max, °C	38.5 (37.9–39)	38.5 (37.9–39)	38.5 (37.7–39)	0.97	
Fever	49 (62.8)	22 (68.7)	32 (69.6)	>0.99	
Hypothermia	4 (5.1)	0 (0)	4 (8.7)	0.14	
SOFA score	6.5 (3.8–11)	8 (5–12.7)	5 (3–11)	0.07	-
White Blood Cells /mm^3^, ×1000	13.4 (9.5–18.1)	13.94 (11.47–19.63)	12.97 (9.25–16.83)	0.69	
Leucopenia	2 (2.6)	1 (3.1)	1 (2.2)	>0.99	
CRP, mg/L	121 (62.7–155)	125 (58–204)	119 (63.2–141)	0.34	
Procalcitonin, μg/L	1.23 (0.34–2.08)	1.51 (0.51–2.94)	1.01 (0.22–2.16)	0.19	
Final model’s accuracy, AUC (95% CI)					**0.71 (0.59–0.83)**

Abbreviations: AGNA, anti-Gram-negative activity; APACHE II, Acute Physiology Assessment and Chronic Health Evaluation; AUC, area under the curve; CI, confidence intervals; CRP, C-reactive protein; ICU, intensive care unit; NA, not applicable; SOFA, sequential organ failure assessment. Apart from the cells where it is otherwise stated, all values are in median (IQR) and *n* (%). * Often two or more combined antibiotics; only antibiotics with Gram-negative activity included. No comparison is feasible as patients were usually receiving more than a single antibiotic; # 24 central venous catheters and 1 peripherally inserted central catheter are included; ** Values in bold represent variables that entered the initial, full multivariate models with response variable the development of colistin-resistant bacteremia; ## Final logistic model for a colistin-resistant event. The explanatory variables included APACHE II score and admission due to infection.

**Table 2:** Microbiology of index culture.

		Overall	Colistin-Resistant Group	Colistin-Sensitive Group
Pathogen *				
*Acinetobacter baumannii*		29	12	17
*Klebsiella pneumoniae*		24	8	16
*Pseudomonas aeruginosa*		10	0	10
*Proteus mirabilis*		6	6	0
*Enterobacter cloace*		4	0	4
*Providencia stuartii*		4	4	0
*Serratia marcescens*		2	2	0
Carbapenem-resistant		57	24	33
	Event > 48 h	47	19	28
	Event < 48 h	10	5	5
Colistin MIC (ng/mL) #				
	=2	-	-	8
	=1	-	-	4
	≤0.5	-	-	34

* *p* < 0.01 (chi-square test); Other pathogens include: *Elizabethkingia meningoseptica*, *E. coli*, *Ochrobactrum anthropi*, *Pseudomonas putida*, *Stenotrophomonas maltophilia*, Sphingomonas paucimobilis. # Plausible only in the presence of colistin susceptibility.

**Table 3:** Factors associated with 28-day mortality.

	Dead (*n* = 21)	Alive (*n* = 57)	*p*-Value #	Hazard Ratio (95% CI) ##
Age	75 (67–79)	62 (47–73)	**<0.01**	
Male	12 (57.1)	39 (68.4)	0.42	
APACHE II	20 (19–25)	17 (12–22)	**0.04**	
CCI	4 (4–5)	2 (1–4)	**0.01**	
SOFA Admission	10 (8–12.2)	7 (4–10)	0.01 **	
Prior ICU admission *	8 (38.1)	19 (33.3)	0.79	
Infectious admission	7 (33.3)	14 (24.6)	0.57	
Medical admission	10 (47.6)	38 (66.7)	0.19	
Immunosuppression	2 (9.5)	7 (14)	>0.99	
Source control	10 (47.6)	20 (35.1)	0.43	
Pitt bacteremia score	4 (4–6)	3 (1–4)	**<0.01**	
Septic shock	20 (95.2)	23 (40.4)	**<0.01**	
Colistin-resistance status			**0.80**	
-Colistin-resistant	8 (38.1)	24 (42.1)		
-Colistin-sensitive	13 (61.9)	33 (57.9)		
Colistin MIC ≤ 0.5	12 (57.1)	32 (56.1)	>0.99	
Empirical colistin for at least 3 days	8 (38.1)	25 (43.9)	0.80	
SOFA index day	13 (11–16)	5 (3–9)	**<0.01** **	1.23 (1.03–1.48)
Temperature index day, °C	38 (36.8–38.5)	38.7 (38.1–39.2)	0.01	
WBC index day, 10^3^/mm^3^, ×1000	13.63 (10.44–17.88)	13.43 (9.31–18.14)	0.99	
CRP index day, mg/L	109.4 (71.83–136.25)	126 (61.08–154.5)	0.53	
Procalcitonin index day, ng/mL	1.33 (1.13–5.63)	0.84 (0.32–1.94)	0.20	
Five-day empirical treatment with colistin	6 (28.6)	20 (35.1)	**0.79**	
Ten-day colistin treatment, post-event	5 (23.8)	16 (28.1)	0.58	
One appropriate drug within 24 h post-event	7 (33.3)	21 (36.8)	>0.99	
One appropriate drug within 48 h post-event	8 (38.1)	25 (43.9)	0.80	
Two appropriate drugs within 24 h post-event	3 (14.3)	7 (12.3)	>0.99	
Two appropriate drugs within 48 h post-event	3 (14.3)	11 (19.3)	0.75	

All values are in median (IQR) and *n* (%). APACHE II, Acute Physiology Assessment and Chronic Health Evaluation; CCI: Charlson comorbidity index; CI, confidence intervals; CRP, C-reactive protein; ICU, intensive care unit; MIC, minimal inhibitory concentration; SOFA, sequential organ failure assessment; WBC; white blood cell count. * 2–12 months before the index admission; # Values in bold represent variables that entered the initial, full multivariate Cox model. ** We considered the index SOFA score as it was more recent and clinically more relevant than the admission score. ## Final Cox proportional hazard model for 28-day mortality. The final model, stratified for age, included one explanatory variable, the index day SOFA score; the concordance index was 0.83 (se = 0.128).

## Data Availability

Data supporting the results can be provided from the corresponding author on request. The data are not publicly available due to privacy policy of the hospital.

## Supplementary Materials

The following are available online at https://www.mdpi.com/article/10.3390/antibiotics11030405/s1, Figure S1: The evolution of the median SOFA score of both study groups every two days, starting from two days before the index day until day 10 post-event, Table S1: Patient characteristics regarding the 0.5 ng/mL threshold for colistin resistance, Table S2: Patients’ characteristics regarding bacteremia acquisition >48 h after the present ICU admission., Table S3: Evolution of clinical and laboratory indices at 48 h intervals, Table S4: Antibiotic treatment, Table S5: Outcomes, Table S6: Factors associated with 28-day mortality in patients with bacteremia acquisition >48 h after ICU admission, Table S7: Patient characteristics in relation to SOFA score on the index day, Table S8: Outcomes of bloodstream infections regarding a 0.5 ng/mL threshold for colistin resistance.

## Abbreviations

APACHE II	Acute Physiology Assessment and Chronic Health Evaluation
AUC	area under the curve
BSI	bloodstream infection
CI	confidence interval
CR	colistin-resistant
CRG	colistin-resistant group
CSG	colistin-sensitive group
CVC	central venous catheter
EUCAST	European Committee On Antimicrobial Susceptibility Testing
GN	Gram-negative
ICU	intensive care unit
IQR	interquartile range
MDR	multi-drug-resistant
SOFA	sequential organ failure assessment
