# Supplementary material for: ICU-Associated Gram-Negative Bloodstream Infection: Risk Factors Affecting the Outcome Following the Emergence of Colistin-Resistant Isolates in a Regional Greek Hospital
Source: Antibiotics (Basel). 2022 Mar 17;11(3):405. doi: 10.3390/antibiotics11030405 (PMC8944566; doi:10.3390/antibiotics11030405)
Supplement: Supplementary file 1 [file antibiotics-11-00405-s001.zip › antibiotics-1621972-supplementary.pdf]

Supplementary Material

Table S1. Patient characteristics regarding the 0.5ng/ml MIC threshold for colistin resistance (sensitivity analysis)

	Overall (n=78)	Colistin-resistant plus(n=44)	Colistin optimally sensitive (n=34)	p-value**	Odds ratio (95% CI) ##
Baseline					
Age, years	66(50.2-76)	69(57.7-78)	62(47.2-73.7)	**0.10**	
Male	51(65.4)	32(72.7)	19(55.8)	**0.15**	
Charlson Comorbidity Index	3(1-5)	4(2-5)	2.5(1-5)	**0.38**	
APACHE II	19(13-24)	20.5(15-24.2)	17(12.2-19.7)	**0.02**	**1.10(1.02-1.20)**
SOFA score	8(5-10)	8.5(6-10)	7.5(3.2-10.7)	**0.26**	
Prior ICU stay	27(34.6)	17(38.6)	10(29.4)	0.47	
Medical patients	48(61.5)	28(63.6)	20(58.8)	0.81	
Immunosuppression	9(11.5)	6(13.6)	3(8.8)	0.72	
Admission due to infection	21(26.9)	8(18.2)	13(38.2)	**0.07**	**0.26(0.08-0.77)**
Antibiotics in the previous 3 months	48(61.5)	28(63.6)	20(58.8)	0.81	
Before the event					
Presence of CVC before the event*	61(78.2)	34(77.2)	27(79.4)	>0.99	
Antibiotics in the ICU	61(78.2)	35(79.5)	26(76.5)	0.79	
Maximum number of drugs with AGNA at any time				0.72	
None given	17(21.8)	9(20.5)	8(23.5)		
Single	31(39.7)	15(34.1)	16(47.1)		
Two	9(11.6)	6(13.6)	3(8.8)		
Three	10(12.8)	6(13.6)	4(11.8)		
Four	11(14.1)	8(18.2)	3(8.8)		
Antibiotic classes / class members #					
Third & fourth generation cephalosporins	26(33.3)	15(34.1)	11(32.4)		
Colistin	28(35.9)	15(34.1)	13(38.2)		
Tigecycline	24(30.8)	15(34.1)	9(26.5)		
Carbapenems	33(42.3)	21(47.7)	12(35.3)		
Aminoglycosides	10(12.8)	6(13.6)	4(11.8)		
Quinolones	13(16.7)	11(25)	2(5.9)		
Ampicillin/sulbactam	15(19.2)	9(20.5)	6(17.6)		
Piperacillin/tazobactam	9(11.5)	5(11.4)	4(11.8)		
Ceftazidime/avibactam	7(9)	5(11.4)	2(5.9)		
Index day				NA	
Event, days	10(6-18)	10(5.7-18)	10.5(6.2-17)	0.69	
Timing of the event				>0.99	
>48 hours stay	67(85.9)	38(86.4)	29(85.3)		
<48 hours stay	11(14.1)	6(13.6)	5(14.7)		
Source				0.96	
Primary	32(41)	19(43.2)	13(38.2)		
Catheter-related ¶	25(32.1)	15(34.1)	10(29.4)		
Urinary	5(6.4)	3(6.8)	2(5.9)		
Intraabdominal	5(6.4)	2(4.5)	3(8.8)		
Surgical site infection	5(6.4)	2(4.5)	3(8.8)		
Lung/pleural empyema	4(5.1)	2(4.5)	2(5.9)		
Bone/joint	2(2.6)	1(2.3)	1(2.9)		
Source control performed	30(38.5)	17(38.6)	13(38.2)	>0.99	
Pitt bacteremia score	3(1-4)	4(1-4.2)	3(1-4)	0.41	
Septic shock	43(55.1)	26(59.1)	17(50)	0.49	
Temperature max, ^{0}C	38.5(37.9-39)	38.5(38-39)	38.5(37.6-39)	0.71	
Fever	49(62.8)	28(63.6)	21(61.8)	>0.99	
Hypothermia	4(5.1)	1(2.3)	3(8.8)	0.31	
SOFA score	6.5(3.8-11)	7.5(4-12.2)	5(3-10.5)	0.15	
White Blood Cells /mm^3, x1000	13.4(9.5-18.1)	13.43(10.13-19.62)	13.44(9.39-16.32)	0.88	
Leucopenia	2(2.6)	1(2.3)	1(2.9)	>0.99	
CRP, mg/l	121(62.7-155)	121(59.2-172.5)	120(71.9-141)	0.67	
Procalcitonin, μg/l	1.23(0.34-2.08)	1.39(0.43-1.96)	0.85(0.21-2.21)	0.35	

Abbreviations: AGNA, anti-Gram-negative activity; APACHE II, Acute Physiology Assessment and Chronic Health Evaluation; CRP, C-reactive protein; CVC, central venous catheter; ICU, intensive care unit; MIC, minimal inhibitory concentration; SOFA, sequential organ failure assessment.
Apart from the cells where it is otherwise stated, all values are in median(IQR) and n(%)
*For at least 48 hours.
Often two or more combined antibiotics; only antibiotics with Gram-negative activity included. No comparison is feasible as patients were usually receiving more than a single antibiotic
¶24 central venous catheters and 1 peripherally inserted central catheter are included
**Values in bold represent variables that entered the initial, full multivariable logistic regression model with response variable the development of colistin-resistant bacteremia at a lower threshold.
##The final model's explanatory variables included APACHE II score and admission due to infection.

Table S2. Patient characteristics only regarding bacteremia acquisition >48 hours after ICU admission (sensitivity analysis).

	**Overall (n=67)**	**Colistin-resistant (n=26)**	**Colistin-sensitive (n=41)**	**p-value****	**Odds ratio (95% CI) ##**
Baseline characteristics					
Age	65(50-77)	72(59-78.7)	62(48-71)	**0.05**	
Male	46(68.6)	19(73.1)	27(65.8)	0.60	
Charlson comorbidity index	3(1-5)	4(2.2-5)	2(1-4)	**0.16**	
APACHE II	19(12.5-22)	21.5(15.5-25)	17(10-19)	**0.002**	**1.14(1.05-1.27)**
SOFA admission	8(5-10)	8.5(6.2-10.7)	7(4-10)	**0.13**	
Prior ICU stay	17(25.4)	6(23.1)	11(26.8)	0.78	
Medical patients	41(61.2)	18(69.2)	23(56.1)	0.32	
Immunosuppression	9(13.4)	6(23.1)	3(7.3)	0.08	
Admission due to infection	14(20.1)	3(11.5)	11(26.8)	**0.22**	**0.29(0.05-1.15)**
Antibiotics in the previous 3 months	37(55.2)	13(50)	24(58.5)	0.62	
Before the event					
Presence of CVC ∗	56(83.6)	22(84.6)	34(82.9)	>0.99	
Antibiotics in the ICU, any	61(91)	24(92.3)	37(90.2)	>0.99	
Maximum number of drugs with AGNA at any time				0.31	
None given	6(9)	2(7.7)	4(9.8)		
Single	31(46.3)	9(34.6)	22(53.6)		
Two	9(13.4)	3(11.5)	6(14.6)		
Three	10(14.9)	5(19.2)	5(12.2)		
Four	11(16.4)	7(26.9)	4(9.8)		
Antibiotic classes / class members #					
Third & fourth generation cephalosporins	26(38.8)	11(42.3)	15(36.6)	0.80	
Colistin	23(34.3)	6(23.1)	17(41.5)	0.19	
Tigecycline	24(35.8)	13(50)	11(26.8)	**0.07**	
Carbapenems	33(49.3)	14(53.8)	19(46.3)	0.62	
Aminoglycosides	10(14.9)	5(19.2)	5(12.2)	0.49	
Quinolones	15(22.4)	8(30.8)	7(17.1)	0.23	
Ampicillin/sulbactam	15(22.4)	8(30.8)	7(17.1)	0.24	
Piperacillin/tazobactam	9(13.4)	4(15.4)	5(12.2)	0.73	
Ceftazidime/avibactam	7(10.4)	4(15.4)	3(7.3)	0.42	
Index Day					
Event, days	12(7-21)	13(9.2-23.5)	11(7-18)	0.20	
Source				>0.99	
Primary	32(47.8)	13(50)	19(46.3)		
Catheter-related ¶	21(31.3)	9(34.6)	12(29.3)		
Urinary	3(4.5)	1(3.8)	2(4.9)		
Intraabdominal	4(6)	1(3.8)	3(7.3)		
Surgical site infection	3(4.5)	1(3.8)	2(4.9)		
Lung/pleural empyema	3(4.5)	1(3.8)	2(4.9)		
Bone/joint	1(1.5)	0(0)	1(2.4)		
Source control	24(35.8)	12(46.2)	12(29.3)	0.20	
Pitt bacteremia score	3(1-4)	4(2-4.7)	3(1-4)	0.23	
Septic shock	35(52.2)	13(50)	22(53.7)	0.81	
Temperature max, ^{0}C	38.6(38.05-39.05)	38.5(38-38.95)	38.7(38.1-39.2)	0.52	
Fever	45(67.2)	16(61.5)	29(70.7)	0.59	
Hypothermia	1(1.5)	0(0)	1(2.4)	>0.99	
White Blood Cells /mm^3, x1000	13.92(10.07-17.23)	14.53(11.54-19.48)	13.43(9.64-16.44)	0.74	
Leucopenia	1(1.5)	1(3.8)	0(0)	>0.99	
CRP, mg/l	123(67.4-151)	125(71.6-151)	123(63.2-151)	0.85	
Procalcitonin, μg/l	1.45(0.33-2.94)	1.48(0.56-2,94)	1.32(0.25-2.94)	0.44	

Abbreviations: AGNA, anti-Gram-negative activity; APACHE II, Acute Physiology Assessment and Chronic Health Evaluation CRP, C-reactive protein; CVC, central venous catheter; ICU, intensive care unit; SOFA, sequential organ failure assessment.

Apart from the cells where it is otherwise stated, all values are in median(IQR) and n(%)

∗ For at least 48 hours;

Often two or more combined antibiotics; only antibiotics with Gram – negative activity included. No comparison is feasible as patients were usually receiving more than a single antibiotic

¶24 central venous catheters and 1 peripherally inserted central catheter are included

**Values in bold represent variables that entered the multivariable logistic regression model with response variable the development of colistin resistant bacteremia

##The final model's explanatory variables included APACHE II score and admission due to infection.

Table S3. Evolution of clinical and laboratory indices at 48-hour intervals

**Day**	**-2**	**0**	**2**	**4**	**6**	**8**	**10**
**WBC, x10^3 , overall***	11.7(9.15-14.8)	13.43(9.53-18.14)	11.8(8.16-16.7)	11(7.9-15)	12(9.1-15)	13(9.65-18)	12.67(8.46-15.78)
-CRG	*11.87(9.45-15.18)*	*13.94(11.49-19.61)*	*12.02(7.58-16.82)*	*11.53(9.18-14.92)*	*11.77(8.84-14.48)*	*11.64(8.96-17.62)*	*14.18(8.76-16.69)*
-CSG	*11.64(9.2-14.06)*	*12.95(9.28-16.50)*	*11.09(8.74-16.52)*	*10.26(7.41-15.38)*	*12.86(9.20-15.08)*	*13.47(10.17-18.78)*	*11.31(8.32-15.58)*
**Temperature, ^{0}C, overall***	37.7(37.2-38.2)	38.5(37.8-39)	37.9(37.4-38.5)	37.7(37-38)	37.7(37-38)	38(37.3-38.2)	37.6(37.2-38.3)
-CRG	*37.7(37.2-38)*	*38.5(38-39)*	*38(37.1-38.5)*	*37.6(37-38.5)*	*37.5(37.2-38.2)*	*37.8(37.3-38.4)*	*37.5(37.2-38.6)*
-CSG	*37.7(37.2-38.2)*	*38.6(37.8-39)*	*37.8(37.5-38.4)*	*37.8(37.4-38.4)*	*37.8(37.4-38.7)*	*37.8(37.3-38.2)*	*37.6(37.2-37.9)*
**CRP mg/l, overall***	80.4(40-141)	120.5(62.7-155)	135(73.4-190)	88.1(49-146)	92.9(49-146)	67(38.85-108)	69.75(34.97-125.5)
-CRG	*62.5(38.3-124.3)*	*125(60.7-198)*	*139(86.2-224)*	*87.7(56.4-145)*	*69.9(43.3-124.2)*	*58.9(36.7-84.8)*	*68.4(36.8-96.7)*
-CSG	*92.8(47.3-141.5)*	*118(65.4-140.5)*	*131.5(66.5-167.5)*	*92.3(45.8-157.7)*	*94.4(24.8-168)*	*86.7(40-143.5)*	*70.9(31-163.7)*
**PCT, ng/ml, overall***	0.70(0.24-1.73)	1.225(0.34-2.08)	1.5(0.72-6.24)	1.21(0.4-2.8)	0.81(0.34-5.06)	0.7(0.415-2.05)	0.75(0.31-3.29)
-CRG	*0.99(0.27-1.71)*	*1.51(0.61-2.45)*	*3.73(1.17-6.9)*	*1.63(0.87-3.77) #*	*0.77(0.53-3.25)*	*0.72(0.37-1.54)*	*0.55(0.26-1.98)*
-CSG	*0.6(0.25-2.33)*	*1.01(0.25-1.92)*	*1.28(0.51-5.96)*	*0.78(0.34-1.54) #*	*0.87(0.34-7.06)*	*0.69(0.62-3.93)*	*0.85(0.44-4.33)*
**SOFA, overall***	5(3-9)	7(4-11)	6(3-11)	6(3-9)	5(2-8)	5(3-10.3)	5(3-11)
-CRG	*6(4.2-11)*	*8(5-12.2)*	*6(4.5-11.5)*	*6(3-9)*	*5(3-7)*	*5(3.2-8)*	*5(4-11)*
-CSG	*5(3-7)*	*5(3-10)*	*5(3-10)*	*4(2.5-10)*	*4(2-10)*	*5(2.2-13.2)*	*5(2-9.5)*

Abbreviations: CRG, colistin-resistant group; CSG, colistin-sensitive group; CRP, C-reactive protein; PCT, procalcitonin; SOFA, sequential organ failure assessment., WBC, white blood cell count.

All values: median(IQR).

*All p-values for parameter decrease between the early and the late days<0.01 (Tukey test, alternative method=”less”)

p-value=0.04, between CRG and CSG.

Table S4. Antibiotic treatment

	Overall (n=78)	Colistin resistant(n=32)	Colistin sensitive(n=46)	p-value
Empirical				
Empirical antibiotic therapy received∗	65(83.3)	28(87.5)	37(80.4)	0.54
One appropriate drug within first 24 hours	28(35.9)	10(31.2)	18(39.1)	0.63
Two appropriate drugs within first 24 hours	10(12.8)	3(9.4)	7(15.2)	0.51
One appropriate drug within first 48 hours	33(42.3)	12(37.5)	20(43.5)	0.65
Two appropriate drugs within first 48 hours	14(17.9)	3(9.4)	10(21.7)	0.22
Empirical colistin#, for at least 5 days	26(33.3)	7(21.9)	19(41.3)	0.09
Targeted				
Number of patients received	66(78.2)	26(81.2)	40(87)	0.54
Time to definite treatment, days	1(0-4)	3(0-5)	0(0-3)	0.07
Number of targeted drugs				0.51
Monotherapy	33(42.3)	15(46.9)	18(39.1)	
Two drug combination	26(33.3)	8(25)	18(39.1)	
Three drug combination	7(9)	3(9.4)	4(8.7)	
Antibiotic de-escalation ¶	6(7.7)	2(6.2)	4(8.7)	>0.99
Total duration of antibiotic treatment	11(8-16)	10.5(8-15.75)	14(11-18)	0.13
Five-day colistin treatment	44(56.4)	15(46.9)	29(63)	0.17
Ten-day colistin treatment	24(30.8)	6(18.7)	18(39.1)	0.08

∗Initiated up to 48 hours post event; #may include the continuation of the drug as targeted therapy up to day 5.
¶Within 5 days

Table S5. Outcomes

	Overall (n=78)	Colistin resistant (n=32)	Colistin sensitive (n=46)	p-value
28-day mortality post event	21(26.9)	8(25)	13(28.3)	0.80
14-day mortality post event	18(23.1)	8(25)	10(21.7)	0.79
ICU mortality	29(37.2)	11(34.4)	18(39.1)	0.85
Hospital mortality	31(39.7)	12(37.5)	19(41.3)	0.82
ICU stay overall	26(13.2-46.5)	31(13.5-51)	23.5(13.7-39.7)	0.32
ICU stay post event	14(6-26.2)	15.5(7-33)	14(5-22.2)	0.25
Recurrent bacteremia	13(16.7)	5(15.6)	8(17.4)	0.88
Secondary bacteremia	19(24.4)	10(31.2)	9(19.6)	0.47
Gram-negative	17(21.8)	8(25)	9(19.6)	
Gram-positive	2(2.6)	2(6.2)	0(0)	
Candida species	0(0)	0(0)	0(0)	
Mechanical ventilation days overall	14.5(3.7-28.2)	17.5(2.5-32)	13(4-23.7)	0.58
Mechanical ventilation days post event	4.5(1-13.5)	5.5(1-16.5)	4(0.2-11.5)	0.64
Renal SOFA 7 days post event	0(0-2)	0.5(0-3.2)	0(0-2)	0.39
Renal SOFA 14 days post event	0(0-3.5)	0(0-2)	0(0-4)	0.94
Renal replacement therapy on day 7 post event	12/56	6/24	6/32	0.74
Renal replacement therapy on day 14 post event	11/42	4/18	7/24	0.73
Renal failure free days post event, days	9(2-18)	8.5(2-23.2)	9(2-17)	0.71
Readmission	5(6.4)	4(12.5)	1(2.2)	0.15

ICU, intensive care unit; SOFA, sequential organ failure assessment

Table S6. Factors associated with 28-day mortality in patients with bacteremia acquisition >48 hours after ICU admission

	**Dead(n=17)**	**Alive(n=50)**	**p-value #**	**Hazard ratio (95% CI) ****
Age	75(67-79)	60(46.2-71.7)	**0.003**	
Male	11(64.7)	35(70)	0.76	
APACHE II	19(18-22)	17(12-22)	0.18	
CCI	4(4-5)	2(0.25-4)	**0.004**	
SOFA Admission	9(8-12)	7(4.2-10)	0.09 ¶	
*Prior ICU admission**	5(29.4)	12(24)	0.75	
Infectious admission	3(17.6)	14(28)	>0.99	
Medical admission	10(58.8)	31(62)	>0.99	
Immunosuppression	2(11.8)	7(14)	>0.99	
Source control	7(41.2)	17(34)	0.77	
Pitt bacteremia score	4(3-6)	3(1-4)	**0.02**	
Septic shock	16(94.1)	19(38)	<0.001	
Colistin resistant	7(41.2)	19(38)	>0.99	
Colistin MIC<=0.5	6(35.3)	23(46)	0.57	
Empirical colistin for at least 3 days	15(88.2)	39(78)	0.49	
SOFA index day	13(11-16)	4.5(3-8.5)	**0.02 ##**	**1.23(1.12-1.34)**
Temperature index day, ^{0}C	38(37.6-38.5)	38.75(38.15-39.2)	0.02	
Hypothermia	1(5.9)	0(0)	0.25	
WBC index day, 10^{3} / mm^{3}, x1000	14.88(11-17.47)	13.44(9.39-16.96)	0.62	
CRP index day, mg/L	123(71.9-151)	124(61.7-151)	0.87	
Procalcitonin index day, ng/mL	1.93(1.32-5.25)	1.23(0.28-2.54)	**0.04**	
Five-day empirical treatment with colistin	10(58.8)	31(62)	>0.99	
Ten-day colistin treatment, post event	4(23.5)	19(38)	0.38	
One appropriate drug within 24h post event	6(35.3)	18(36)	>0.99	
One appropriate drug within 48h post event	7(41.2)	21(42)	>0.99	
Two appropriate drugs within 24h post event	2(11.8)	6(12)	>0.99	
Two appropriate drugs within 48h post event	2(11.8)	10(20)	0.72	

All values are in median(IQR) and n(%)
APACHE II, Acute Physiology Assessment and Chronic Health Evaluation; CCI: Charlson comorbidity index; CI, confidence intervals; CRP, C-reactive protein; ICU, inyensive care unit; MIC, minimal inhibitory concentration; SOFA, sequential organ failure assessment; WBC; white blood cell count.
*2-12 months before the index admission
# Values in bold, represent variables that entered the initial, full multivariate Cox model with response variable the 28-day mortality.
¶ We only considered the index SOFA score as it was more recent and clinically more relevant than the admission score
**Final Cox proportional hazard model where the explanatory variables were age and the index day SOFA score; the likelihood ratio test was the concordance index was 0.80 (se = 0.063).

Table S7. Patient characteristics in relation to SOFA score on the index day (high and low according to median)

	Overall (n=78)	Increased SOFA (n=40)	Lower SOFA (n=38)	p-value**	Multivariable logistic regression analysis##
Baseline characteristics					
Age	66(50.2-76)	72.5(60.5-79)	63(47.7-71.7)	**0.04**	1.03(1.0-1.07)
Male	51(65.4)	27(67.5)	24(63.2)	0.81	
Charlson comorbidity index	3(1-5)	4(2-5)	2(0.2-4)	**0.08**	
APACHE II	19(13-24)	18(12-21)	19.5(16-24)	**0.02**	
SOFA admission	8(5-10)	10(7-11.2)	6(3-9)	**0.001**	1.28(1.11-1.51)
Prior ICU stay	27(34.6)	18(45)	9(23.7)	**0.06**	3.98(1.33-13.4)
Medical patients	48(61.5)	23(57.5)	25(65.8)	0.49	
Immunosuppression	9(11.5)	6(15)	3(7.9)	0.48	
Admission due to infection	21(26.9)	10(25)	11(28.9)	0.80	
Antibiotics in the previous 3 months	48(61.5)	26(65)	22(57.9)	0.64	
Before the event					
Presence of CVC *	61(78.2)	34(85)	27(71.1)	0.17	
Antibiotics in the ICU, any	61(78.2)	29(72.5)	32(84.2)	0.28	
Maximum number of drugs with AGNA at any time				0.28	
None given	17(21.8)	11(27.5)	6(15.8)		
Single	31(39.7)	11(27.5)	20(52.6)		
Two	9(11.6)	5(12.5)	4(10.5)		
Three	10(12.8)	5(12.5)	5(13.2)		
Four	11(14.1)	8(20)	3(7.9)		
Antibiotic classes / class members #					
Third & fourth generation cephalosporins	26(33.3)	11(34.4)	15(32.6)	0.34	
Colistin	28(35.9)	15(37.5)	13(34.2)	0.82	
Tigecycline	24(30.8)	13(32.5)	11(28.9)	0.81	
Carbapenems	33(42.3)	17(42.5)	16(42.1)	>0.99	
Aminoglycosides	10(12.8)	7(17.5)	3(7.9)	0.31	
Quinolones	13(16.7)	7(17.5)	6(15.8)	>0.99	
Ampicillin/sulbactam	15(19.2)	8(25)	7(15.2)	>0.99	
Piperacillin/tazobactam	9(11.5)	4(12.5)	5(10.9)	0.73	
Ceftazidime/avibactam	7(9)	3(7.5)	4(10.5)	0.71	
Index Day					NA
Event, days	10(6-18)	8.5(4-16.2)	12(7.2-22.7)	0.03	
Colistin resistant	32(41)	19(47.5)	13(34.2)	0.26	
Timing of the event				0.19	
>48 hours stay	67	32(80)	35(92.1)		
<48 hours stay	11	8(20)	3(7.9)		
Source				0.19	
Primary	32(41)	14(35)	18(47.4)		
Catheter-related¶	25(32.1)	12(30)	13(34.2)		
Urinary	5(6.4)	2(5)	3(7.9)		
Intraabdominal	5(6.4)	2(5)	3(7.9)		
Surgical site infection	5(6.4)	5(12.5)	0(0)		
Lung/pleural empyema	4(5.1)	3(7.5)	1(2.6)		
Bone/joint	2(2.6)	2(5)	0(0)		
Source control	30(38.5)	15(37.5)	15(39.5)	>0.99	
Pitt bacteremia score	3(1-4)	4(4-6)	1(0-3)	0.0002	
Septic shock	43(55.1)	37(92.5)	6(15.8)	<0.001	
Temperature max, ^{0}C	38.5(37.9-39)	38.3(37.7-38.8)	38.7(38.1-39.3)	0.04	
Fever	49(62.8)	22(55)	27(71.1)	0.17	
Hypothermia	4(5.1)	4(12.5)	0(0)	0.12	
White Blood Cells /mm^3, x1000	13.4(9.5-18.1)	14.88(11.1-19.76)	12.88(8.74-16.46)	0.22	
Leucopenia	2(2.6)	2(5)	0(0)	0.49	
CRP, mg/l	121(62.7-155)	121.5(71.67-173.75)	118.5(58.58-138.5)	0.20	
Procalcitonin, μg/l	1.23(0.34-2.08)	1.69(0.43-4.09)	0.72(0.27-1.5)	0.11	

Abbreviations: AGNA, anti-Gram-negative activity; APACHE II, Acute Physiology Assessment and Chronic Health Evaluation; CRP, C-reactive protein; CVC, central venous catheter; ICU, intensive care unit; SOFA, sequential organ failure assessment.
Apart from the cells where it is otherwise stated, all values are in median(IQR) and n(%).
*For at least 48 hours.
Often two or more combined antibiotics; only antibiotics with Gram – negative activity included. No comparison is feasible as patients were usually receiving more than a single antibiotic
¶24 central venous catheters and 1 peripherally inserted central catheter are included
** Values in bold represent variables that entered the initial, full multivariable logistic regression model with response variable the higher half of the index day SOFA score.
The final model's explanatory variables included age, prior ICU stay, and admission SOFA score**.**

Table S8. Outcomes of bloodstream infections regarding a 0.5ng/ml threshold for colistin resistance

	**Overall (n=78)**	**Colistin resistant plus (n=44)**	**Colistin optimally sensitive (n=34)**	**p-value**
28-day mortality post event	21(26.9)	12(27.3)	9(26.5)	>0.99
14-day mortality post event	18(23.1)	12(27.3)	6(17.6)	0.42
ICU mortality	29(37.2)	17(38.6)	12(35.3)	0.82
Hospital mortality	31(39.7)	18(40.9)	13(38.2)	>0.99
ICU stay overall	26(13.2-46.5)	28(12-47.5)	23.5(17-42.2)	0.85
ICU stay post event	14(6-26.2)	12.5(6.7-28.2)	15.5(5-23)	0.78
Recurrent bacteremia	13(16.7)	6(13.6)	7(20.6)	0.54
Secondary bacteremia	19(24.4)	12(27.3)	7(20.6)	0.51
Gram-negative	17(21.8)	10(22.7)	7(20.6)	
Gram-positive	2(2.6)	2(4.5)	0(0)	
Candida species	0(0)	0(0)	0(0)	
Mechanical ventilation days overall	14.5(3.7-28.2)	15.5(2-28.7)	14(4.2-26.2)	0.84
Mechanical ventilation days post event	4.5(1-13.5)	4.5(0.2-12)	4.5(1-16.7)	0.74
Renal SOFA 7 days post event	0(0-2)	1(0-3.2)	0(0-1.2)	0.13
Renal SOFA 14 days post event	0(0-3.5)	0(0-2.5)	0(0-3)	0.44
Renal replacement therapy on day 7 post event (n=56)	12	8	4	
Renal replacement therapy on day 14 post event (n=42)	11	6	5	
Renal failure free days post event, days	9(2-18)	7(1.7-18.5)	11(3.2-17.7)	0.78
Readmission	5(6.4)	5(11.4)	0(0)	0.06

Abbreviations: ICU, intensive care unit; SOFA, sequential organ failure assessment

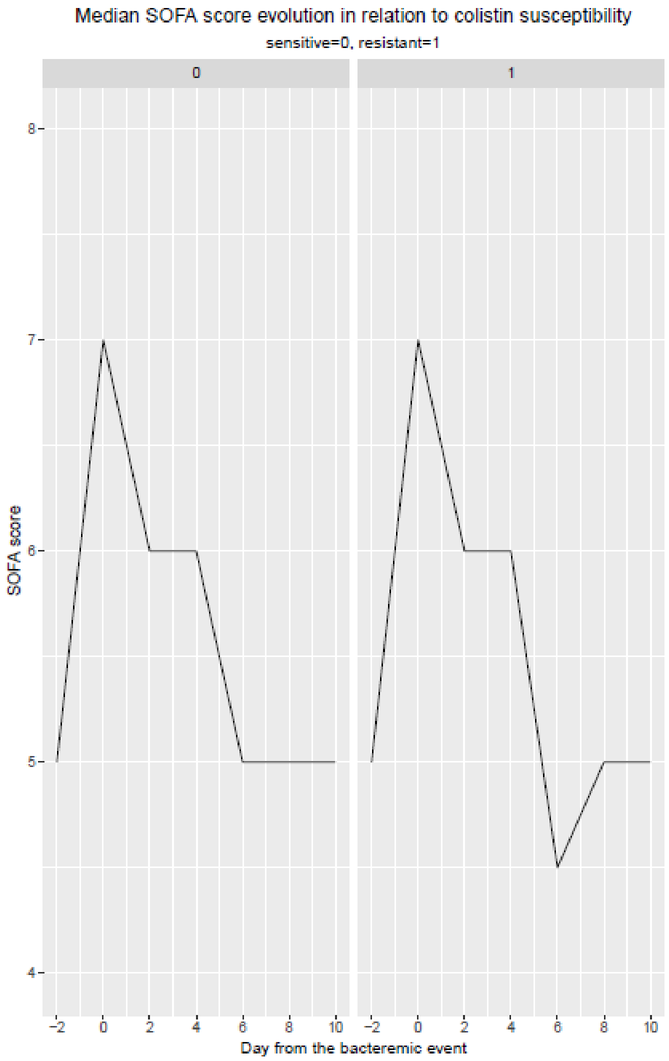

Figure S1. The evolution of the median SOFA score of both study groups every two days, starting from two days before the index day until day 10 post-event.
